# Inflammatory bowel disease and renal disorders: from clinical associations to shared mechanisms and management strategies

**DOI:** 10.3389/fimmu.2026.1812348

**Published:** 2026-04-29

**Authors:** Shi Zheng, Hui Wen, Wenmeng Yin, Dongping Chen, Xiaolin Zhong

**Affiliations:** 1 Department of Gastroenterology, The Affiliated Hospital of Southwest Medical University, Luzhou, China; 2 Department of Nephrology, Shuguang Hospital Affiliated to Shanghai University of Traditional Chinese Medicine, Shanghai, China

**Keywords:** gut-kidney axis, inflammatory bowel disease, kidney disease, microbiota, systemic inflammation

## Abstract

A growing body of evidence indicates a strong relationship between inflammatory bowel disease (IBD) and a range of renal conditions, including chronic kidney disease, glomerulonephritis, tubulointerstitial disorders, nephrolithiasis, and secondary (AA) amyloidosis, which are rare yet severe systemic complications. In this review, we integrated clinical and epidemiological data to clarify the pathophysiological mechanisms underlying the gut–kidney axis. Existing evidence was critically appraised with the observation that, although associative data are plentiful, causal pathways in humans remain incompletely understood. We describe how dysbiosis of the gut microbiota, breakdown of the intestinal barrier, and altered microbial metabolites (e.g., trimethylamine N-oxide [TMAO] and short-chain fatty acids [SCFAs]) drive systemic inflammation and contribute to renal injury. Newer findings point to intestinal lymphatic dysfunction as a crucial intermediary. Injury to the proteinuric kidney triggers lymphangiogenesis within the gut and reshapes the composition of the lymph, thereby facilitating the systemic delivery of pro-inflammatory mediators, including IsoLG-modified apolipoprotein AI and Th17 cells, that worsen renal damage, thus establishing a self-reinforcing pathological loop. Nonetheless, evidence supporting the lymphatic pathway currently relies mainly on animal studies, with limited human validation. We also examined the contribution of conserved pathways such as IL-11-driven fibrosis, GSDMD-mediated pyroptosis, and free light chain toxicity. From a translational standpoint, these overlapping pathways represent promising therapeutic targets, although further testing is required to establish their clinical applicability. Such mechanistic insights underscore the importance of vigilant renal monitoring in individuals with IBD and careful management of drug-induced nephrotoxicity. Strategies that target these shared mechanisms, whether through restoration of the microbiota, modulation of lymphatic function, or precision immunomodulation, herald a new frontier for therapies aimed at dual-organ protection.

## 1 Introduction

Inflammatory bowel disease (IBD), encompassing Crohn’s disease (CD)and ulcerative colitis (UC), is a chronic relapsing disorder of the gastrointestinal tract driven by aberrant mucosal immunity (1). Increasingly, IBD is recognized as a systemic inflammatory state with extraintestinal manifestations (EIMs) spanning the skin, joints, eyes, and urinary tract (2). Despite affecting nearly one-quarter of patients, renal involvement remains underappreciated relative to other EIMs (3). This clinical blind spot is compounded by the absence of standardized renal surveillance protocols, the non-specific presentation of early urological symptoms, and growing concerns regarding the nephrotoxic potential of advanced immunotherapies (4). Consequently, a proactive, mechanism-informed approach to renal risk assessment in IBD is urgently required.

Robust epidemiological data firmly associate IBD with a spectrum of renal pathologies, including chronic kidney disease, glomerulonephritis, tubulointerstitial nephritis, nephrolithiasis, and secondary (AA) amyloidosis (5–8). This gut–kidney crosstalk is mechanistically anchored in intestinal dysbiosis and barrier compromise, which initiate remote organ injury (9, 10). Microbial metabolic shifts—characterized by elevated trimethylamine N-oxide (TMAO) and diminished short-chain fatty acids (SCFAs)—establish a biochemical conduit between mucosal inflammation and renal damage (11, 12). Emerging evidence further implicates intestinal lymphatic dysfunction as a pivotal mediator: proteinuric injury drives lymphangiogenesis and alters lymphatic flow, facilitating the systemic dissemination of pro-inflammatory mediators (e.g., IsoLG-modified apolipoprotein AI, cytokines, and immune cells) that amplify renal pathology and perpetuate a self-sustaining inflammatory loop (13–15).

Beyond microbial translocation, convergent immunopathogenic pathways drive tissue injury across the intestinal and renal compartments (16, 17). Interleukin-11 (IL-11) has emerged as a master regulator of profibrotic signaling, bridging epithelial stress responses with fibroblast activation in both organs (18). Concurrently, gasdermin D-mediated pyroptosis amplifies local inflammation and tissue destruction in IBD and glomerular disease (19). Humoral dysregulation further links these systems, with aberrant B-cell-derived free light chains exerting dual tropism for the gut and kidneys (20). Genetic susceptibility underscores this shared pathogenesis; for instance, DNASE1L3 loss-of-function variants predispose to both early-onset IBD and lupus nephritis (21). In severe, refractory cases, sustained systemic inflammation culminates in secondary (AA) amyloidosis, wherein serum amyloid A deposits predominantly in the kidneys, epitomizing the systemic toll of chronic IBD activity (8).

Deciphering these overlapping pathogenic axes is critical for optimizing clinical management. Key priorities include implementing risk-stratified renal surveillance, mitigating drug-induced nephrotoxicity (notably from 5-aminosalicylates) (22), and addressing systemic complications such as inflammation-driven anemia (23). Furthermore, targeting shared pathways—through microbiome modulation, lymphatic functional restoration, or precision immunotherapies—holds promise for dual gut–kidney therapeutic benefit (24). This review synthesizes contemporary evidence spanning epidemiology, mechanistic biology, and clinical management. By delineating the bidirectional pathophysiology of IBD-associated renal injury, we aim to establish a framework for integrated, interdisciplinary care and to highlight novel therapeutic targets that transcend organ-specific boundaries.

## 2 In-depth clinical and epidemiological evidence

### 2.1 Phenotype-dependent CKD risk and renal burden in IBD

Clinical and epidemiological evidence establishes inflammatory bowel disease as an independent risk factor for chronic kidney disease (CKD), with progression to renal impairment representing a key clinical manifestation. Large-scale analyses confirm that patients with IBD face a significantly elevated CKD risk relative to the general population, persisting after adjustment for conventional renal risk factors. A meta-analysis encompassing >100,000 IBD cases and 760,000 controls reported a pooled odds ratio (OR) of 1.59 (95% CI 1.31–1.93), approximating a 60% risk elevation. Cohort data from the UK Biobank corroborate this association, demonstrating a hazard ratio (HR) of 1.32 (95% CI 1.15–1.51) for incident CKD (25). This relationship remains consistent irrespective of outcome ascertainment—whether via diagnostic coding (OR 1.70, 95% CI 1.33–2.19) or eGFR-based criteria (OR 1.36, 95% CI 1.33–1.64) (26)—reinforcing IBD as a substantive contributor to renal disease burden. Risk stratification within the IBD population reveals marked heterogeneity. Crohn’s disease (CD) confers a higher burden of CKD and end-stage renal disease (ESRD) than ulcerative colitis (UC) (27). Disease severity further modulates risk: extra-intestinal manifestations, prior intestinal resection, and prolonged disease duration independently predict accelerated CKD progression (28). Age exerts a distinct influence: although relative risk peaks in adolescence (adjusted HR 7.88 [95% CI 2.56–24.19] at age 16), absolute incidence rises with advancing age, with the adjusted HR attenuating to 1.13 (95% CI 1.01–1.25) by age 77 (5).

The disproportionate renal risk in CD versus UC warrants mechanistic interrogation beyond phenotypic severity. In a cohort of 3,557 CD patients, renal biopsy—performed in 0.56%—confirmed IgA nephropathy (IgAN) in 70% of cases, whereas IgAN incidence in UC remained low at 0.9% (29, 30). This disparity likely reflects convergent pathophysiological pathways. First, CD-characteristic transmural inflammation more profoundly compromises intestinal barrier integrity, facilitating systemic translocation of bacterial products (e.g., lipopolysaccharides) and dietary antigens (31, 32). This exposure may aberrantly activate mucosal plasma cells, driving overproduction of galactose-deficient IgA1 (Gd-IgA1), a central pathogenic mediator in IgAN (33). Second, CD-associated stricturing or penetrating disease frequently necessitates intestinal resection, predisposing to chronic dehydration, electrolyte imbalance, and enteric hyperoxaluria—each independently promoting nephrolithiasis and CKD (28, 29). Third, therapeutic heterogeneity may partially confound observed risks: anti-TNFα agents, preferentially used in CD, have been linked to rare cases of renal IgA vasculitis, whereas UC management more commonly involves 5-aminosalicylates, which carry a well-characterized risk of tubulointerstitial nephritis that may mask or mimic primary glomerular pathology (22, 34, 35). Collectively, these insights indicate that elevated renal risk in CD arises from the convergence of heightened mucosal immune activation, surgical sequelae, genetic susceptibility, and differential drug exposure. The multifactorial pathways linking IBD to CKD—encompassing chronic systemic inflammation and metabolic dysregulation—are detailed in subsequent sections, which delineate the full pathological spectrum of renal involvement.

### 2.2 Spectrum of IBD-associated glomerular diseases

Among IBD-associated glomerulopathies, IgA nephropathy (IgAN) predominates, accounting for approximately 75% of biopsy-proven cases in Crohn’s disease and 25% in ulcerative colitis. Longitudinal data indicate that IBD diagnosis typically precedes renal involvement by a median of nine years, implicating chronic mucosal inflammation as a driver of delayed nephropathy (6, 29). This clinical association is underpinned by shared genetic architecture: a Mendelian randomization study of 86,640 individuals of European ancestry established a causal link between IBD susceptibility and IgAN risk (OR 1.78; 95% CI 1.45–2.19), with CD4^+^ T-cell CD4 expression emerging as a significant genetically correlated trait (OR 2.72; 95% CI 1.10–6.72) (30). Clinically, patients present with hypertension and substantial proteinuria, and renal biopsies typically demonstrate active inflammatory lesions. Although long-term renal survival broadly mirrors that of primary IgAN, approximately 16.7% of cases progress to end-stage renal disease (ESRD) or experience a >50% decline in eGFR, highlighting substantial prognostic heterogeneity (29). Beyond IgAN, active intestinal inflammation precipitates a diverse array of glomerular pathologies (6, 31, 32). IBD is characterized by sustained systemic immune activation, marked by elevated TNF-α, IL-6, IL-1β, and BAFF (34–36), alongside aberrant B-cell differentiation, dysregulated IgA class switching, and chronic T-cell activation (37–39). These immunological perturbations directly compromise glomerular integrity. Biopsy registries and case series document a spectrum of lesions during disease flares, including focal segmental glomerulosclerosis (FSGS), minimal change disease (MCD), and crescentic glomerulonephritis (40–42). Proposed mechanisms encompass cytokine-mediated podocyte effacement, T-cell-driven alterations in glomerular permselectivity, and systemic inflammatory amplification. Critically, effective control of intestinal inflammation frequently induces remission of FSGS and MCD, underscoring a direct immunological axis between gut pathology and podocyte dysfunction (42, 43). Hence, IBD-associated glomerular diseases exhibit marked phenotypic heterogeneity, spanning common immune-complex-mediated injuries to rare autoimmune glomerulopathies. This diversity reflects partially elucidated, yet fundamentally shared, immunopathogenic pathways bridging intestinal and renal compartments. The observed clinical responsiveness to gut-directed therapies further suggests that biologic agents targeting specific inflammatory cytokines may hold therapeutic potential for concurrent autoimmune renal disease. Nevertheless, definitive mechanistic delineation and large-scale prospective trials are requisite to validate these pathways and to establish evidence-based, organ-agnostic treatment paradigms for IBD-related nephropathy.

### 2.3 IBD-associated tubulointerstitial diseases

Tubulointerstitial nephritis (TIN) represents a major renal complication in IBD, broadly dichotomized into pharmacologically induced and primary immune-mediated forms. 5-aminosalicylic acid (5-ASA), a cornerstone of mild-to-moderate IBD management (44), is well-documented to cause chronic interstitial nephritis (45). Although incidence remains low (0.3% annually), clinical sequelae can be severe, with progression to end-stage renal disease (ESRD) necessitating renal replacement therapy in select cases (46). Toxicity typically manifests insidiously after a median exposure of 3 years, presenting with microscopic hematuria, microalbuminuria, sterile pyuria, and progressive eGFR decline (22). Consequently, routine renal surveillance via urinalysis and eGFR assessment is imperative, though optimal monitoring intervals remain contested owing to heterogeneous international guidelines (4). Pharmacogenomic evidence implicates the HLA-DRB1*03:01 allele as a susceptibility marker for 5-ASA nephrotoxicity (22). Beyond 5-ASA, calcineurin inhibitors (e.g., cyclosporine, tacrolimus) employed in refractory disease carry substantial nephrotoxic potential (47). These agents induce acute tubulointerstitial injury and electrolyte derangements, with chronic exposure driving interstitial fibrosis and irreversible functional decline (47, 48). Historical precedents further underscore this iatrogenic axis; gold salts, once utilized for rheumatologic conditions, were documented to concurrently provoke colitis and nephropathy, illustrating how certain immunomodulators can simultaneously compromise gastrointestinal and renal homeostasis (49).

Systemic immune dysregulation inherent to IBD also directly mediates primary (non–drug-induced) tubulointerstitial injury. TIN is increasingly recognized as an extraintestinal manifestation, with renal biopsies frequently revealing granulomatous inflammation that substantiates an immune-driven pathogenesis (50). Epidemiological analyses confirm a significantly elevated prevalence of IBD among biopsy-confirmed TIN cohorts relative to the general population (50). Mechanistically, aberrantly produced B-cell-derived free light chains (FLCs) not only propagate intestinal inflammation but also exert direct cytotoxic effects on proximal tubular epithelium, thereby driving intrinsic tubulointerstitial damage independent of pharmacological exposure (20). Clinically, this subclinical injury can be detected via urinary biomarkers; α1-microglobulin and cystatin C demonstrate high sensitivity for early tubular dysfunction, enabling risk stratification even before overt renal impairment manifests (51). In summary, tubulointerstitial pathology in IBD encompasses both drug-induced toxicity and primary immune-mediated injury. Mitigating renal risk necessitates vigilant surveillance, particularly in patients receiving nephrotoxic regimens, alongside heightened recognition of primary TIN as a bona fide extraintestinal manifestation. Integrating pharmacogenomic screening and sensitive tubular biomarkers into routine care will be pivotal for preserving renal function and optimizing long-term clinical trajectories in this vulnerable population.

### 2.4 Renal amyloidosis in IBD

Beyond common glomerular and tubulointerstitial pathologies, chronic uncontrolled inflammation in IBD can rarely culminate in secondary (AA) amyloidosis, a severe systemic complication (8). This condition arises from sustained acute-phase responses that drive overproduction of serum amyloid A (SAA), leading to extracellular deposition of insoluble fibrils in multiple organs, predominantly the kidneys. Although overall prevalence remains low (0.53% of 10,000 IBD patients), risk is markedly phenotype-dependent: CD confers a 13-fold higher amyloidosis burden than UC (1.05% vs 0.08%), implicating transmural inflammation as a key pathogenic driver (52). Renal involvement typically presents as nephrotic syndrome with progressive functional decline, yet diagnosis is frequently delayed owing to insidious onset; notably, ~15% of cases lack overt proteinuria or elevated serum creatinine at initial assessment, necessitating heightened vigilance in high-risk cohorts (52). Definitive diagnosis requires biopsy demonstrating Congo red–positive amyloid deposits, most commonly obtained from renal tissue (52, 53). Prognosis remains guarded: AA amyloidosis is associated with increased acute tubular necrosis, accelerated progression to advanced CKD and ESRD, and reduced survival (54). Management prioritizes aggressive suppression of intestinal inflammation to curtail hepatic SAA synthesis (53). Although no curative therapy exists for established deposits, combination regimens incorporating anti-TNF agents and colchicine have demonstrated potential to halt disease progression (52, 55). Collectively, renal amyloidosis represents a rare but devastating endpoint of sustained systemic inflammation in IBD. The disproportionate morbidity in CD underscores the imperative of achieving and maintaining deep remission—particularly in patients with long-standing active disease—to mitigate extraintestinal complications and preserve renal function.

### 2.5 Urological complications in IBD

Nephrolithiasis constitutes the most significant urological complication associated with IBD, an association documented since the 1970s (56, 57). Historical studies initially reported a substantially higher prevalence than in the general population, while recent prospective cohorts have provided precise quantitative estimates. A survey of 2,323 Swiss patients demonstrated imaging-detected nephrolithiasis prevalence rates of 4.6% in CD and 3.0% in UC (58), whereas another cohort (n = 3,104) reported incidence rates of 6.7% and 6.0%, respectively (59). Furthermore, a large-scale meta-analysis involving over 13 million individuals indicated a pooled prevalence of approximately 6.3% (95% CI 4.8–8.3%), with a consistently higher risk in CD compared with UC (7.9% vs 5.6%) (60). A Danish nationwide cohort of 75,236 patients further confirmed this elevated risk, showing a 2.27-fold increased hazard for urinary stones (95% CI 2.17–2.38). Notably, this risk was already elevated prior to formal IBD diagnosis (OR 1.42; 95% CI 1.34–1.50) (61). Despite these robust associations, clinical diagnosis is frequently delayed because stone-related symptoms are often atypical or masked by predominant bowel complaints, sometimes resulting in significant renal impairment at presentation. The risk of nephrolithiasis is not uniformly distributed and is closely linked to specific clinical determinants. Multivariate analyses identify male sex, active disease, history of intestinal surgery, NSAID use, and physical inactivity as significant independent risk factors (58). Among these, extensive bowel resections (e.g., total proctocolectomy) markedly elevate risk by inducing chronic low urine volume and hypocitraturia (62). Calcium oxalate and uric acid stones predominate in this population, with their pathogenesis tightly coupled to enteric hyperoxaluria and disrupted mucosal barrier function (63).

**Figure 1:**
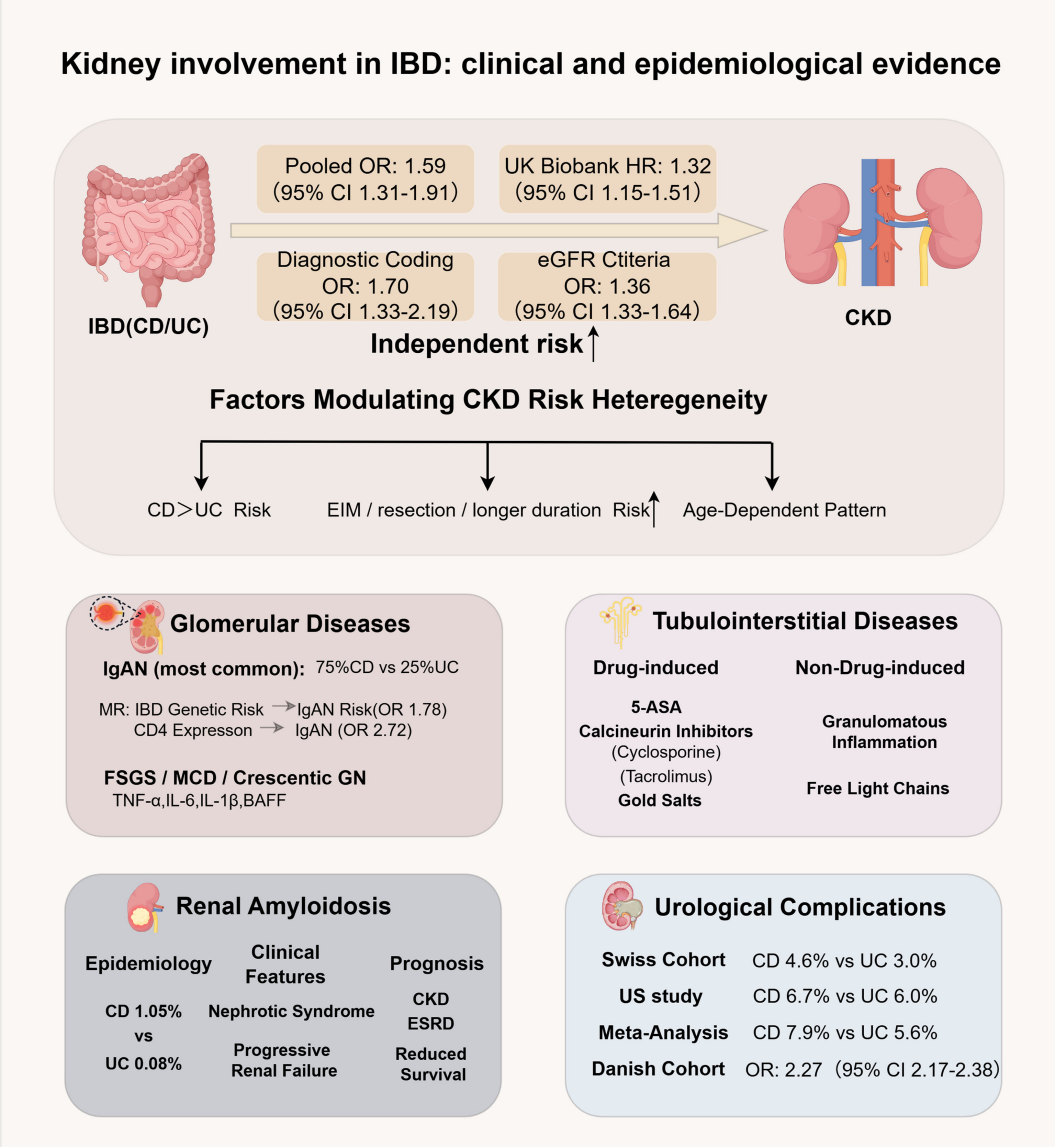
Kidney involvement in IBD: clinical and epidemiological evidence. This image summarizes the clinical and epidemiological evidence linking IBD to various renal disorders, including an increased risk of chronic kidney disease (CKD), predominance of Crohn’s disease (CD) and of ulcerative colitis (UC) in most renal complications, and a diverse pathological spectrum. This includes glomerular diseases (IgA nephropathy, FSGS, and MCD), tubulointerstitial nephritis (drug-induced and primary), AA amyloidosis, and urological complications such as nephrolithiasis. The key risk modulators include disease phenotype, extraintestinal manifestations (EIMs), disease duration, and age. Abbreviations: 5-ASA, 5-aminosalicylic acid; MR, Mendelian randomization.

In addition to nephrolithiasis, other urological complications, although less frequent, exhibit well-defined epidemiological profiles. Enterovesical fistulas represent a severe penetrating complication of CD, occurring in approximately 2–4% of patients (64, 65). Additionally, the relative risk of renal and urinary tract malignancies in IBD is approximately fivefold higher than in the general population (66) (65). Risk factor analyses indicate that smoking independently escalates urological cancer risk in CD (67), whereas thiopurine therapy increases the incidence of urinary tract neoplasms by approximately threefold (68). Advanced age further compounds overall malignancy susceptibility (69). Although the association between bladder cancer and IBD remains debated, an upward trend in the CD subgroup warrants sustained clinical attention (70). In summary, the markedly elevated burden of urological complications underscores the imperative for long-term, systematic surveillance in this population. The comprehensive spectrum of renal involvement in IBD, spanning glomerular disease, tubulointerstitial nephritis, amyloidosis, and urological sequelae, is summarized in **Figure 1**. The robust epidemiological associations described herein—particularly the higher renal risk in CD versus UC, the delayed onset of IgAN following IBD diagnosis, and the increased risk of CKD even in young patients—collectively suggest that chronic transmural intestinal inflammation drives sustained systemic immune activation. However, epidemiological data alone cannot establish causality or elucidate precise biological pathways. The following sections therefore transition from descriptive epidemiology to mechanistic exploration of the gut–kidney axis, focusing on how intestinal dysbiosis, barrier dysfunction, lymphatic remodeling, and shared inflammatory cascades translate epidemiological risk into organ injury.

## 3 Convergent pathophysiological networks in gut–kidney comorbidity

**Figure 2:**
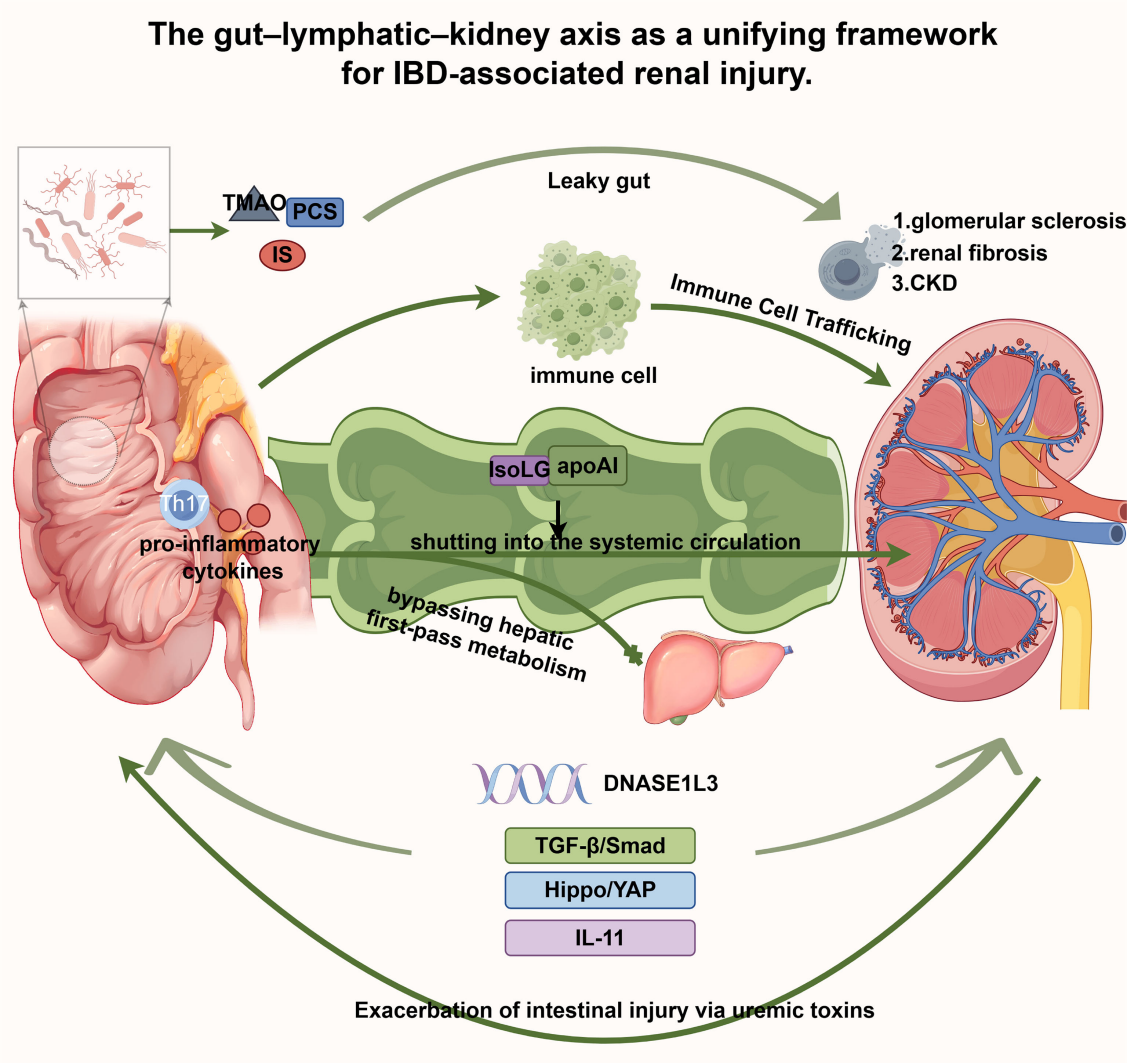
The gut–lymphatic–kidney axis as a unifying framework for IBD-associated renal injury. This schematic integrates the key pathophysiological mechanisms linking intestinal inflammation to renal injury, including gut barrier dysfunction, microbial metabolite translocation, intestinal lymphatic remodeling, and downstream fibrotic pathways. In IBD, intestinal barrier disruption (“leaky gut”) allows translocation of microbial metabolites (TMAO, IS, PCS) and activated immune cells into the intestinal wall, contributing to subsequent renal injury. The intestinal lymphatic system undergoes pathological remodeling and serves as a conduit for systemic dissemination. Pathogenic mediators, including IsoLG-apoAI, cytokines, and immune cells, are shuttled into circulation, bypassing hepatic first-pass metabolism. In the kidney, gut-derived signals activate multiple pro-fibrotic pathways (TGF-β/Smad, Hippo/YAP, IL-11) and inflammasome-mediated pyroptosis, driving glomerular sclerosis, interstitial fibrosis, and progression to CKD. Genetic susceptibility (e.g., DNASE1L3 deficiency) is further linked to intestinal and renal pathologies. Declining renal function leads to uremic toxin accumulation, which exacerbates intestinal barrier disruption and establishes a vicious self-reinforcing cycle. Abbreviations: CKD, chronic kidney disease; IBD, inflammatory bowel disease; IL-11, interleukin-11; IS, indoxyl sulfate; IsoLG-apoAI, isolevuglandin-modified apolipoprotein AI; PCS, p-cresyl sulfate; TGF-β, transforming growth factor-β; TMAO, trimethylamine Noxide; YAP, Yes-associated protein.

In addition to clinical associations, a network of shared pathophysiological mechanisms solidifies the link between IBD and renal disorders. As illustrated in **Figure 2**, these mechanisms form an integrated network rather than an isolated pathway. The following sections discuss the interconnected pathways.

### 3.1 Microbial dysbiosis and metabolite-driven gut–kidney crosstalk

The gut–kidney axis has emerged as the central theoretical framework for elucidating the intrinsic link between IBD and renal diseases, emphasizing the bidirectional regulatory relationship between gut health and renal function (71, 72). The core driver of this dialog is the gut microbial equilibrium. Gut dysbiosis is a hallmark of chronic inflammatory conditions such as IBD (73). This imbalance disrupts the intestinal mucosal barrier integrity, leading to increased gut permeability or a “leaky gut” (12, 74). At the molecular level, “leaky gut” primarily manifests as a functional breakdown of tight junction proteins (75). In the inflammatory milieu of IBD, pro-inflammatory cytokines (TNF-α, IL-6, IL-1β) markedly downregulate the expression and spatial organization of key tight junction proteins (occludin, claudin-1, ZO-1), dismantling epithelial continuity (76, 77). Conversely, microbial metabolites such as butyrate serve as critical barrier stabilizers (68). Butyrate engages GPR109A on intestinal epithelial cells and inhibits histone deacetylases (HDAC), thereby restoring tight junction protein expression and promoting mucosal repair (78).

Microbial metabolic activity further dictates host pathophysiology through the systemic dissemination of bioactive metabolites (79). Trimethylamine N-oxide (TMAO), an extensively characterized deleterious metabolite, functions as a circulating molecular conduit that translates dysbiotic signals into renal inflammatory cascades (80, 81). TMAO exacerbates renal injury through convergent mechanisms. First, it triggers PANoptosis in renal tubular epithelial cells by concurrently activating apoptotic (caspase-8), pyroptotic (NLRP3, caspase-1, GSDMD), and necroptotic (ZBP1/RIP3/MLKL) pathways (82). Second, TMAO binds and activates the endoplasmic reticulum stress kinase PERK (EIF2AK3), selectively engaging the unfolded protein response. This axis upregulates FoxO1 and drives fibroblast proliferation and collagen synthesis via mTOR/Akt signaling, directly precipitating renal fibrosis (83, 84). Third, TMAO induces ferroptosis—an iron-dependent, lipid peroxidation-mediated cell death—in tubular epithelium, accelerating CKD progression (85). Fourth, it amplifies oxidative stress via NADPH oxidase 2 (NOX2) activation and stimulates NF-κB signaling, fostering a senescence-associated secretory phenotype (SASP) (86, 87). Finally, TMAO exacerbates pre-existing injury by potentiating crystal deposition, autophagic dysfunction, and inflammation in models such as hyperoxaluria-induced nephropathy (88). Collectively, these pathways establish TMAO as a central molecular hub linking dysbiosis to renal fibrosis and functional decline (89).

Concurrently, the uremic toxins indoxyl sulfate (IS) and p-cresyl sulfate (PCS) have emerged as pivotal mediators within the gut–kidney axis (90, 91). Derived from microbial catabolism of aromatic amino acids (tryptophan and tyrosine), these compounds are normally cleared renally. In CKD, however, systemic accumulation directly induces tubular apoptosis, drives interstitial fibrosis, and activates oxidative stress pathways that reciprocally compromise intestinal tight junctions (90–92). IS and PCS mediate renal injury through multifaceted mechanisms. First, they suppress the Nrf2/Keap1 antioxidant pathway, exacerbating oxidative stress and tubular damage (93). Second, IS activates the aryl hydrocarbon receptor (AhR) to initiate NF-κB signaling, amplifying IL-6 and TNF-α production (94), while PCS promotes renal inflammation via monocyte activation (95). Third, IS drives fibrogenesis through the AhR/Akt axis, upregulating c-Myc and EGFR to stimulate pathological epithelial proliferation (94). Additionally, IS impairs mitochondrial quality control by suppressing mitophagy via the IRF1/DRP1 axis, precipitating intestinal barrier failure and endotoxin translocation that further aggravates renal interstitial fibrosis (96). Beyond direct cytotoxicity, IS and PCS disrupt renal transport dynamics; high-protein diets upregulate OAT1 and OAT3, increasing secretory load until transporter exhaustion and functional decline ensue (97). Notably, PCS exhibits markedly reduced renal clearance compared with IS (median 6.8 vs. 17.7 mL/min), predisposing to greater accumulation and more severe nephrotoxicity (98). These interconnected pathways forge a self-perpetuating “gut–kidney–gut” vicious cycle. Dysbiosis and mucosal barrier disruption initially elevate circulating precursors of uremic toxins, which subsequently accumulate systemically as renal clearance capacity declines. This mounting metabolite burden then reciprocally compromises intestinal epithelial integrity, further exacerbating microbial dysbiosis and amplifying systemic inflammation (71, 99). Such a feed-forward loop constitutes a fundamental pathophysiological engine that concurrently drives the progression of both intestinal and renal disease.

### 3.2 Intestinal lymphatic dysfunction

**Figure 3:**
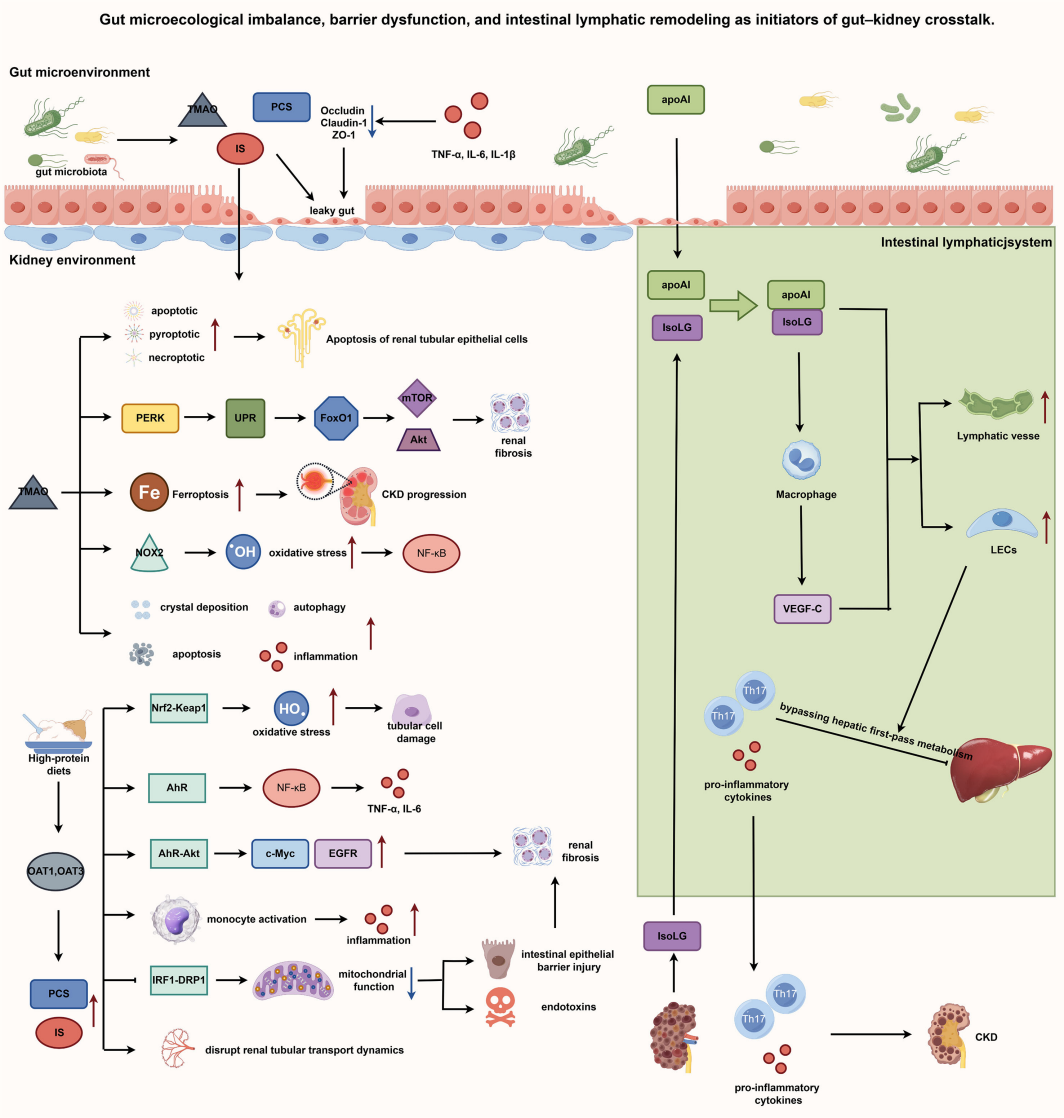
Gut microecological imbalance, barrier dysfunction, and intestinal lymphatic remodeling as initiators of gut–kidney crosstalk. Integration of the mechanisms of gut dysbiosis, barrier disruption, intestinal lymphatic remodeling, and downstream renal injury pathways. In IBD, gut microbiota dysbiosis and pro-inflammatory cytokines (TNF-α, IL-6, IL-1β) downregulate tight junction proteins (occludin, claudin-1, ZO-1), leading to increased intestinal permeability (“leaky gut”). This facilitates the translocation of harmful microbial metabolites (TMAO, IS, and PCS) into circulation. TMAO induces PANoptosis (apoptotic, pyroptotic, and necroptotic signaling), PERK-mediated unfolded protein response (UPR) leading to fibrosis, and NOX2-dependent oxidative stress. IS and PCS impair the Nrf2/Keap1 antioxidant pathway, activate AhR/NF-κB signaling, disrupt renal tubular transport via OAT1/OAT3, and impair mitochondrial function through the IRF1/DRP1 axis. Gut-derived mediators that reach the kidney activate multiple injurious pathways. The intestinal lymphatic system undergoes pathological remodeling, with increased lymph flow and altered lymph composition. IsoLG modifies apoAI to form IsoLG–apoAI, which activates lymphatic endothelial cells (LECs) and promotes VEGF- secretion by macrophages. These mediators, along with Th17 cells and pro-inflammatory cytokines, are efficiently shuttled into systemic circulation, bypassing hepatic first-pass metabolism. These mechanisms drive tubular cell damage, inflammation, and CKD progression. Abbreviations: AhR, aryl hydrocarbon receptor; apoAI, apolipoprotein AI; CKD, chronic kidney disease; DRP1, dynamin-related protein 1; IL, interleukin; IRF1, interferon regulatory factor 1; IS, indoxyl sulfate; IsoLG, isolevuglandin; LEC, lymphatic endothelial cell; NF-κB, nuclear factor κB; NOX2, NADPH oxidase 2; Nrf2, nuclear factor erythroid 2-related factor 2; OAT, organic anion transporter; PCS, p-cresyl sulfate; PERK, protein kinase R-like endoplasmic reticulum kinase; TMAO, trimethylamine N-oxide; TNF-α, tumor necrosis factor α; UPR, unfolded protein response; VEGF-C, vascular endothelial growth factor C; ZO-1, zonula occludens-1.

Recent investigations have redefined the intestinal lymphatic system as a pivotal mediator of gut–kidney axis communication (15). Beyond canonical roles in lipid absorption and immune surveillance, intestinal lymphatics function as a potent amplifier and dissemination conduit for systemic inflammation and remote organ injury (13). This gut–lymphatic–kidney axis operates through reciprocal pathological remodeling, wherein injury in either compartment induces profound structural and functional alterations in the contralateral lymphatic network. In proteinuric kidney disease models, marked intestinal lymphangiogenesis, a fivefold increase in lymph flow, and dysregulated lymphatic lipid composition (notably high-density lipoprotein and apolipoprotein A-I [apoA-I]) are consistently observed. Central to this remodeling is isolevuglandin (IsoLG), a reactive dicarbonyl generated by renal oxidative stress. IsoLG covalently modifies intestinally derived apoA-I to form IsoLG-apoA-I (14). This adduct not only abrogates physiological apoA-I function but acquires potent pathogenic properties. IsoLG-apoA-I directly augments lymphatic contractility, activates lymphatic endothelial cells, and stimulates macrophage-derived VEGF-C secretion. Consequently, modified biomolecules, activated immune subsets, and pro-inflammatory cytokines are efficiently routed into systemic circulation via remodeled lymphatic vessels, circumventing hepatic first-pass clearance (14). This “lymphatic stealth” pathway enables the unimpeded delivery of pro-inflammatory mediators to distant target organs, establishing a self-sustaining pathogenic circuit. Initial renal injury drives intestinal IsoLG accumulation and lymphatic remodeling, which subsequently amplifies systemic inflammatory trafficking, exacerbates nephropathy, and perpetuates tissue damage. Accordingly, the intestinal lymphatic network functions as a high-capacity conduit for gut-derived noxious signals, providing a novel mechanistic framework for understanding how IBD precipitates extraintestinal renal injury. This paradigm further establishes a compelling rationale for developing lymphatic-directed therapeutics to interrupt disease propagation. The integrated mechanisms of microbial dysbiosis, barrier failure, and lymphatic remodeling are summarized in **Figure 3**.

### 3.3 Convergent inflammatory signaling and aberrant immune cell trafficking

The comorbidity of inflammatory bowel disease (IBD) and renal pathology is underpinned by shared systemic inflammation and immune dysregulation. IBD constitutes a systemic inflammatory state, with circulating mediators exerting distal organ effects (3). Central to this axis is the NLRP3 inflammasome, a cytosolic multiprotein complex that integrates endogenous danger signals (e.g., TMAO, urate crystals, ROS) and microbial cues (100). Within the gut–kidney axis, intestinal barrier disruption and dysbiosis trigger NLRP3 activation in both compartments (101). This initiates gasdermin D (GSDMD) cleavage and pyroptotic cell death, culminating in robust IL-1β and IL-18 secretion, with signal amplification mediated by caspase-8-dependent pathways (102). Consequently, NLRP3 functions as a pivotal molecular node that transduces intestinal metabolic, microbial, and danger signals into remote renal inflammation, serving as a convergent mechanism for gut-driven nephropathy (101). Aberrant immune cell homing further bridges intestinal and renal pathology. In IBD, activated Th17 cells upregulate gut-tropic homing receptors, notably CCR6 and α4β7 integrin (103, 104). Under conditions of systemic inflammation, renal endothelium expressing cognate ligands (CCL20 and MAdCAM-1) can ectopically recruit these circulating Th17 cells (105). Subsequent renal infiltration drives local tissue injury via IL-17A secretion. This misdirected homing extends beyond the Th17 lineage, establishing a broader paradigm of shared immunological trafficking.

**Figure 4:**
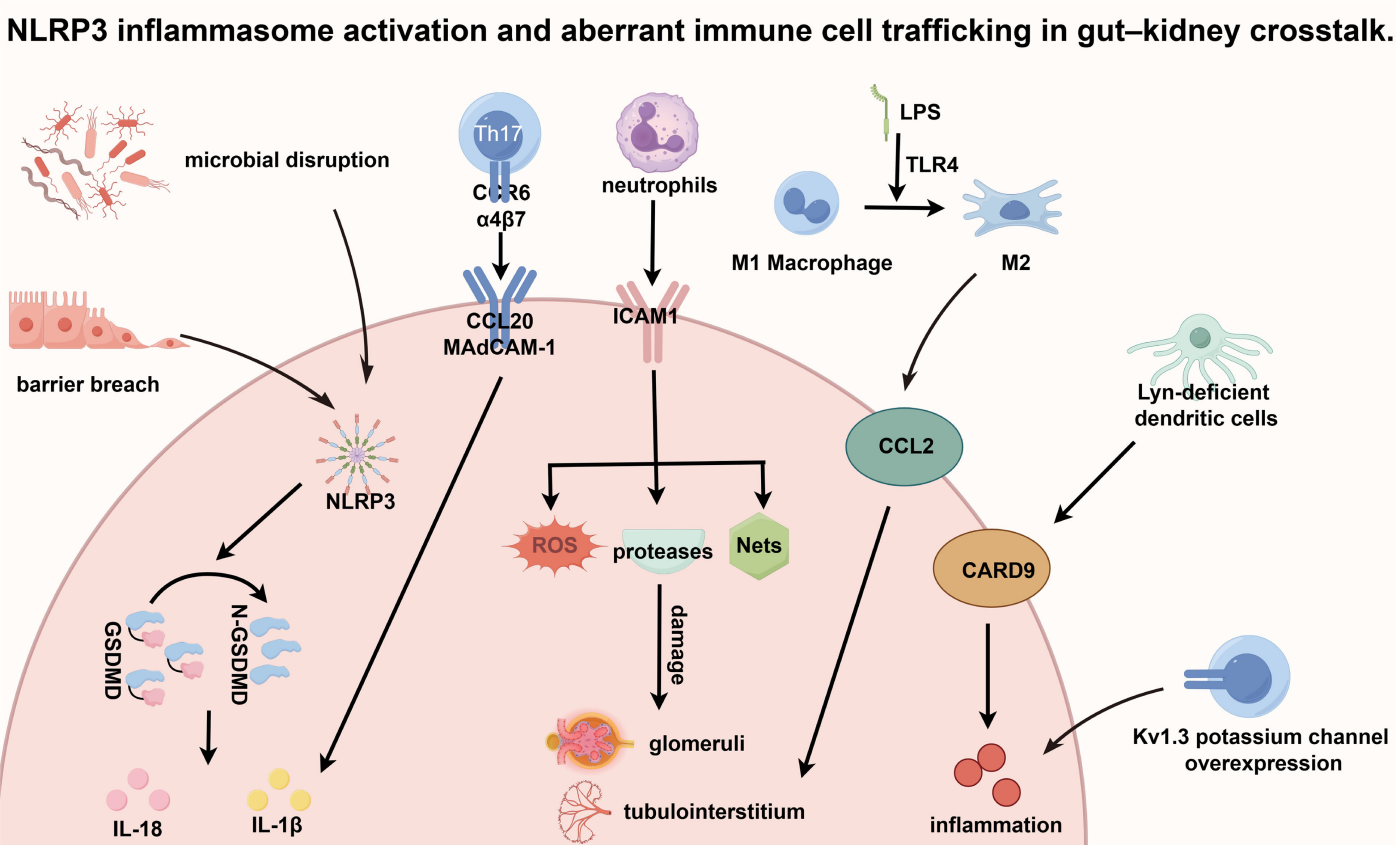
NLRP3 inflammasome activation and aberrant immune cell trafficking in gut–kidney crosstalk. Two interconnected mechanisms that translate gut-derived inflammatory signals into renal injury: inflammasome-mediated inflammation and aberrant immune cell homing. Gut-derived signals, including microbial disruption, barrier breach, and lipopolysaccharide (LPS), activate the NLRP3 inflammasome via Toll-like receptor 4 (TLR4) signaling. This triggers gasdermin D (GSDMD) cleavage, inducing pyroptosis and release of IL-1β and IL-18. Macrophages polarize toward a pro-inflammatory M1 phenotype, amplifying local inflammation. In the inflamed gut, Th17 cells highly express the homing receptors CCR6 and α4β7 integrin. Under systemic inflammation, the kidneys upregulate the corresponding ligands (CCL20, MAdCAM-1) and adhesion molecules (ICAM 1), leading to the erroneous recruitment of gut-derived lymphocytes. Additional immune cells, including neutrophils, which release reactive oxygen species (ROS), proteases, and neutrophil extracellular traps (NETs)contribute to tissue injury. The infiltrating immune cells perpetuate glomerular and tubulointerstitial inflammation. Dysregulated dendritic cell signaling (e.g., Lyn-deficient or CARD9-dependent pathways) and Kv1.3 potassium channel overexpression in activated T lymphocytes further promote sustained immune activation and pathological infiltration, driving progressive renal injury. Abbreviations: CARD9, caspase recruitment domain-containing protein 9; GSDMD, gasdermin D; ICAM1, intercellular adhesion molecule 1; IL, interleukin; LPS, lipopolysaccharide; MAdCAM-1, mucosal addressing cell adhesion molecule-1; NETs, neutrophil extracellular traps; NLRP3, NLR family pyrin domain-containing 3; ROS, reactive oxygen species; TLR4, toll-like receptor 4.

Neutrophils and macrophages amplify this crosstalk through synchronized recruitment and activation. In ulcerative colitis, epithelial MAPK signaling and ICAM1-mediated adhesion orchestrate neutrophil influx (106, 107) Patients with concomitant IBD and chronic kidney disease exhibit parallel upregulation of ICAM1 and neutrophil infiltration in colonic and renal tissues, which correlate positively (107). Infiltrating neutrophils release ROS, proteases, and neutrophil extracellular traps that directly compromise glomerular and tubulointerstitial integrity (108, 109). Concurrently, macrophage phenotypic plasticity dictates tissue repair versus fibrosis. In ulcerative colitis, lipopolysaccharide drives TLR4-dependent M1 polarization, while macrophage apoptosis inhibitor (AIM) sustains inflammation by impairing apoptotic clearance (110). In Crohn’s disease-associated IgA nephropathy, renal macrophage density surpasses that of primary disease and closely parallels glomerulosclerosis and interstitial fibrosis (108). These cells are predominantly recruited from the circulation via chemokine gradients, particularly CCL2 (111). Dendritic cells (DCs) further link intestinal dysregulation to autoimmune nephropathy. Lyn-deficient DCs exhibit CARD9-dependent Toll-like receptor hyperactivation, concurrently driving colitis and systemic autoimmunity (112). Given that CARD9 polymorphisms are implicated in IgA nephropathy, this pathway represents a conserved mechanism whereby aberrant DC activation propagates concurrent gut and renal injury (112–114). Additionally, Kv1.3 potassium channel overexpression in activated T lymphocytes sustains pathological immune infiltration in both IBD and CKD (115). Collectively, these synchronized trafficking and activation pathways elucidate why renal deterioration in IBD frequently mirrors intestinal disease activity, underscoring the immunological unity of the gut–kidney axis. The mechanistic convergence of NLRP3 inflammasome signaling and dysregulated immune cell homing is delineated in **Figure 4**.

### 3.4 Genetic susceptibility and shared molecular signaling

Convergent genetic architectures underpin the comorbidity of inflammatory bowel disease (IBD) and select autoimmune nephropathies, indicating shared heritable risk factors (116). Epidemiological data further substantiate this nexus, demonstrating robust co-occurrence between IBD and psoriasis—a systemic inflammatory disorder independently associated with chronic kidney disease (CKD) progression (117, 118). Such phenotypic clustering implicates conserved genetic pathways that orchestrate analogous immunopathogenic cascades across organ systems. At the molecular level, DNASE1L3 deficiency provides direct evidence of a genetic gut–kidney axis. DNASE1L3 encodes a critical deoxyribonuclease, and its functional loss drives a broad autoimmune spectrum characterized by impaired clearance of immunogenic nucleic acid complexes (119). Biallelic pathogenic variants in DNASE1L3 frequently manifest as juvenile-onset systemic lupus erythematosus (jSLE), with lupus nephritis representing a severe, prognostically significant complication. Notably, integrated clinical and systematic analyses have identified early-onset IBD as a distinct phenotype in DNASE1L3-deficient individuals, directly linking a monogenic defect to concurrent intestinal and renal inflammatory pathology (120). This deficiency exhibits a distinctive immunological signature: in contrast to the constitutive type I interferonopathy characteristic of Aicardi–Goutières syndrome, affected patients demonstrate transient interferon pathway activation that resolves upon clinical remission. The defining clinical and molecular characteristics of DNASE1L3 deficiency as a paradigm for gut–kidney genetic crosstalk are delineated in **Supplementary Table S1**.

### 3.5 Cytokine networks in gut–kidney fibrogenesis

Fibrosis constitutes the maladaptive terminus of chronic inflammation and aberrant tissue repair, representing a convergent pathological endpoint in both IBD (manifesting as intestinal stricturing and functional impairment) and CKD (progressing inexorably to end-stage renal disease) (121, 122). Central to this fibrogenic cascade is the TGF-β/Smad axis. Persistent inflammatory cues drive robust TGF-β secretion in both compartments (123, 124). Ligand–receptor engagement triggers Smad2/3 phosphorylation, Smad4 complex formation, and nuclear translocation, thereby upregulating profibrotic effectors such as α-smooth muscle actin (α-SMA) and fibrillar collagens (125). While this core circuitry is conserved, tissue-specific contexts dictate its execution. In the intestine, chronic inflammation couples with metabolic–epigenetic regulation—exemplified by GCGR/GLP1R-mediated histone lactylation—to drive direct activation of resident fibroblasts (126) (126). Conversely, renal fibrosis responds to diverse metabolic and hemodynamic insults, wherein lactate-driven histone lactylation predominantly fuels epithelial–mesenchymal transition (EMT) (127). These contextual divergences highlight organ-specific vulnerabilities amenable to targeted intervention.

In recent years, the Hippo/YAP signaling pathway has garnered significant attention for its role in sensing mechanical cues and regulating fibrosis (128). In intestinal strictures and renal interstitial fibrosis, increased tissue stiffness inhibits the Hippo pathway, leading to activation of its downstream effector YAP (Yes-associated protein) and its nuclear translocation (129). Nuclear YAP engages TEAD family factors to directly induce profibrotic gene programs and synergize with TGF-β/Smad signaling, thereby potently driving myofibroblast expansion and matrix accumulation (129). Organ-specific topologies persist: renal YAP/TAZ activation predominates in tubular epithelial and interstitial fibroblasts, whereas intestinal activation localizes to mesenteric-derived fibroblasts. Upstream regulation similarly diverges, with TGF-β/Smad dominating renal fibrogenesis and TNF-α/mechanical stress prevailing in the gut. Therapeutically, the apparent absence of kidney-specific fibroblast regulatory nodes (e.g., PGI2/PTGIR) may necessitate broader YAP/TAZ suppression in renal disease.

Beyond these classic pathways, IL-11 has emerged as a pivotal upstream orchestrator of cross-organ fibrosis. Epithelial injury induces IL-11 upregulation, which propagates fibroblast activation and matrix deposition via ERK-dependent signaling (18). Despite this shared function, cellular origins and downstream consequences exhibit marked organ specificity. In the kidney, IL-11 derives from fibroblasts and injured tubular epithelial cells (TECs), driving EMT through the STAT3/ERK1/2–metadherin axis (130). Macrophage-derived IL-11 further acts in a paracrine manner on fibroblasts to accelerate interstitial fibrosis in diabetic nephropathy (131). Functionally, renal IL-11 promotes tubular atrophy and collagen expansion, with expression levels strongly correlating with serum creatinine (132). Critically, IL-11 neutralization in Alport syndrome reverses EMT and extends survival (133). In contrast, intestinal IL-11 is predominantly fibroblast-restricted, governed by GLIS3-dependent transcriptional networks (co-regulating IL-11 and CCL20) that lack TEC involvement and operate via a macrophage-to-fibroblast relay (134, 135). Clinically, intestinal IL-11 correlates with stricturing (B2) phenotypes in Crohn’s disease, and circulating levels may stratify surgical risk (135). Its pathogenicity centers on extracellular matrix remodeling and collagen crosslinking rather than direct epithelial toxicity (134). Targeted IL-11 blockade thus represents a promising dual-organ antifibrotic strategy. The shared and organ-specific features of these fibrotic pathways are summarized in **Table 1**. These mechanistic insights provide a robust foundation for clinical management. The following section translates these shared pathways into practical strategies for risk stratification, monitoring, and therapeutic interventions.

**Table 1:** Shared and organ-specific mechanisms in fibrosis.

Pathway	Role in fibrosis	Organ-specific features
TGF-β/Smad	Core, canonical pro-fibrotic pathway driving myofibroblast activation and ECM deposition	Intestine: Driven primarily by chronic inflammation; linked to metabolic–epigenetic regulation (e.g., histone lactylation). Kidney: Triggered by diverse stimuli (e.g., hypertension, diabetes); involves prominent epithelial-mesenchymal transition (EMT).
Hippo/YAP	Mechanosensitive pathway: nuclear YAP synergizes with TGF-β/Smad to amplify fibrosis	Intestine: Activation occurs mainly in mesentery-derived fibroblasts; driven by TNF-α and mechanical stress. Kidney: Activation occurs in tubular epithelial cells and interstitial fibroblasts; may require broader YAP/TAZ inhibition due to lack of fibroblast-specific compensatory pathways.
IL-11	Upstream master regulator of fibrosis; drives fibroblast activation via ERK signaling	Intestine: Restricted primarily to fibroblast populations; regulated by GLIS3-driven gene networks; correlates with fibrostenosing Crohn’s disease. Kidney: Expressed by both fibroblasts and injured tubular epithelial cells (TECs); induces EMT; involves macrophage-to-fibroblast delivery; correlates with serum creatinine.

## 4 Clinical management and therapeutic strategies

The elucidated mechanisms, including the emerging “gut–lymphatic–kidney axis,” inform a paradigm shift in clinical management, moving from reactive care to proactive, precision strategies grounded in the gut–kidney axis.

### 4.1 Risk-stratified monitoring and early detection of gut–kidney crosstalk in IBD

The markedly increased susceptibility to renal complications in inflammatory bowel disease (IBD) warrants a transition from uniform screening to risk-adapted surveillance. Although all patients require baseline assessment, monitoring intensity must be calibrated to specific high-risk determinants, including Crohn’s disease with stricturing or penetrating phenotypes, extra-intestinal manifestations, prior intestinal resection, refractory inflammation, concurrent nephrotoxin exposure (e.g., NSAIDs, proton pump inhibitors), and hypoalbuminemia (5, 28, 136). Standardized surveillance mandates baseline serum creatinine (for eGFR estimation) and urinalysis for proteinuria and hematuria in all individuals (136, 137). For patients prescribed 5-aminosalicylates, a graduated schedule is indicated: monthly serum creatinine for 3 months, quarterly for 9 months, and biannually or annually thereafter (138, 139). High-risk cohorts warrant intensified surveillance at 6–12-month intervals, independent of therapeutic regimen (136, 139). The incorporation of novel tubular injury biomarkers is strongly justified to enable the earlier detection of subclinical renal injury. Urinary liver-type fatty acid–binding protein (L-FABP) and neutrophil gelatinase–associated lipocalin (NGAL) provide sensitive indicators of tubular stress before serum creatinine elevation, thereby refining early risk stratification (140, 141). Structural and functional assessment via imaging further complements biochemical monitoring. Baseline renal ultrasonography remains essential to exclude anatomical abnormalities, whereas shear-wave elastography enables non-invasive quantification of cortical stiffness as a surrogate for interstitial fibrosis, enhancing longitudinal prognostic tracking (142). Emerging perspective: Given that renal dysfunction can perpetuate systemic pathology by compromising intestinal barrier integrity, microbiome homeostasis, and lymphatic drainage, future surveillance frameworks should incorporate evaluation of intestinal lymphatic architecture and function—through advanced imaging or fluid biomarker profiling—to elucidate gut–kidney crosstalk in high-risk populations (13). **Table 2** summarizes this risk-stratified monitoring paradigm.

**Table 2:** Clinical management paradigm for IBD-associated kidney injury.

Domain	Core strategy	Key clinical actions
Risk monitoring	Risk-adapted surveillance	High-risk groups: CD with complications, EIMs, persistent activity, nephrotoxic drug use. Schedule: Baseline eGFR/urinalysis for all; on mesalazine: monitor at 1, 3, then 6–12 monthly; high-risk: monitor every 6–12 months. Emerging tools: Novel biomarkers (L-FABP/NGAL) for subclinical injury.
Drug management	Agent-specific precision	5-ASA: Strict adherence to monitoring; risk of chronic interstitial nephritis. Immunosuppressants: Cyclosporine-nephrotoxic; azathioprine/MTX-dose adjust in renal impairment. Biologics: Vedolizumab/ustekinumab have favorable renal safety. JAK inhibitors: Dose reduction in renal impairment (infection risk).
Shared complication	Anemia of inflammation management	Diagnosis (inflammation context): Ferritin <100 μg/L or 100-300 μg/L with TSAT <20%. First-line: Intravenous iron (rapid high-dose repletion). Emerging: HIF-PH inhibitors targeting core anemic mechanism.

### 4.2 Risk−stratified management of IBD therapy−associated nephrotoxicity

Effective IBD management necessitates balancing disease control with mitigation of treatment−associated renal risks (138). Precision strategies encompass proactive risk stratification, mechanism−guided drug selection, and protocol−driven surveillance. Aminosalicylates (5−ASA/mesalazine) carry a low but clinically significant risk of chronic interstitial nephritis progressing to end−stage renal disease; adherence to structured monitoring (section 4.1) is therefore critical, as injury may persist post−cessation (46, 138, 143, 144). Cyclosporine presents a dual risk profile: acute, dose−dependent nephrotoxicity (typically reversible) versus chronic interstitial fibrosis with prolonged exposure. Though thiopurines and methotrexate lack direct nephrotoxicity, both require dose attenuation in renal impairment to prevent severe systemic toxicity, notably myelosuppression (145–147). Among advanced therapeutics, anti−TNFα agents exhibit minimal direct renal toxicity but can rarely induce immune−mediated pathology such as acute interstitial nephritis or lupus−like glomerulonephritis. Conversely, vedolizumab and ustekinumab demonstrate favorable renal safety without pharmacokinetic adjustment. JAK inhibitors similarly possess low intrinsic nephrotoxicity, yet dose reduction remains standard in moderate−to−severe renal impairment to mitigate infection and off−target effects (145, 148). Integrating therapeutic drug monitoring (TDM) for thiopurines and anti−TNF agents is essential for precision dosing, optimizing therapeutic windows while minimizing adverse events.

### 4.3 Targeting inflammation-driven anemia in IBD and CKD

Anemia of inflammation (AI) and concomitant iron deficiency are highly prevalent in IBD and CKD, substantially impairing quality of life and predicting adverse clinical outcomes (149, 150). Diagnostic precision is confounded by inflammation-driven hyperferritinemia; consequently, functional iron deficiency necessitates adjusted thresholds: serum ferritin <100 μg/L, or 100–300 μg/L with transferrin saturation (TSAT) <20% (151, 152). Given compromised gastrointestinal absorption during active flares, intravenous (IV) iron is the preferred first-line therapy (153), particularly for patients with active mucosal inflammation, moderate-to-severe CKD, or refractory oral iron intolerance (154). Next-generation IV formulations, including ferric carboxymaltose and ferric derisomaltose, enable rapid, high-dose administration (1,000–2,000 mg per infusion), facilitating efficient iron repletion with a superior safety profile and minimal gastrointestinal toxicity relative to legacy preparations (154, 155). Beyond iron repletion, hypoxia-inducible factor prolyl hydroxylase (HIF-PH) inhibitors (for example, roxadustat) represent a mechanistic paradigm shift (156). By stabilizing HIF, these compounds upregulate endogenous erythropoietin synthesis and enhance iron mobilization, directly counteracting the inflammatory suppression of erythropoiesis (157). While firmly established in CKD, their therapeutic utility in IBD-associated AI warrants further clinical validation (158).

### 4.4 Novel therapies targeting shared mechanisms

Elucidating shared gut–immune–kidney pathways reveals therapeutic strategies capable of concurrent multi-organ protection (71) (**Table 3**). Microbiota-directed interventions, notably fecal microbiota transplantation (FMT), can rebalance the gut–kidney axis by lowering uremic toxins (for example, IS and PCS) in CKD and suppressing mucosal inflammation in IBD (159, 160). Translation requires standardized donor screening, validated long-term efficacy and safety protocols for immunocompromised patients. Next-generation biologics expand this paradigm through engineered probiotics (such as SYNB1020 for ammonia/arginine metabolism), targeted phage therapy against specific pathobionts (for example, AIEC), and postbiotic supplementation to deliver beneficial metabolites like short-chain fatty acids (161–163). Phytochemicals with microbiota-modulating properties also show promise. The isoquinoline alkaloid berberine exerts multi-target effects in preclinical models by modulating microbial composition, attenuating inflammation and preserving epithelial barrier integrity (164, 165). Likewise, rhein (a rhubarb-derived metabolite) exhibits anti-inflammatory and antifibrotic activity in renal pathology while ameliorating experimental colitis (166, 167). These agents bridge traditional pharmacology and mechanism-based therapy, meriting rigorous clinical evaluation.

**Table 3:** Emerging therapies targeting shared gut-kidney pathways.

Therapeutic target	Intervention	Key mechanism/benefit
Microbiota	FMT/Engineered microbes	Restore gut–kidney homeostasis; reduce uremic toxins and inflammation.
	Natural compounds (berberine, rhein)	Modulate microbiota; anti-inflammatory and barrier-protective effects.
Immunity and fibrosis	IL-11 inhibition	Simultaneously block fibrotic pathways in both gut and kidney.
	GSDMD inhibition	Suppress pyroptosis to control severe inflammation.
	SGLT2 inhibitors	Reno-protective and anti-fibrotic effects (established in CKD).
Lymphatic system	Anti-lymphangiogenesis	Block pathological lymphatic remodeling to limit systemic spread of gut-derived inflammatory mediators.
	Dicarbonyl scavengers	Neutralize IsoLG, prevent formation of pathogenic IsoLG-apoAI adducts.
	Lymph-directed drug delivery	Targeted delivery to gut lymphatics, minimizing renal exposure.

Direct targeting of shared immune and fibrotic cascades offers a complementary avenue. Neutralizing IL-11, a master regulator upregulated in both intestinal and renal fibrosis, could concurrently mitigate pathological scarring (168). Similarly, inhibiting GSDMD-mediated pyroptosis curbs cross-organ inflammation, with repurposed agents such as disulfiram under active investigation (169). A critical hurdle for both strategies is achieving cell-type specificity to preserve host immune competence. SGLT2 inhibitors, already validated for anti-inflammatory and antifibrotic effects in non-diabetic CKD, may extend renal and cardiovascular protection to patients with comorbid IBD (170, 171). Prospective trials are warranted to establish their efficacy and safety in this distinct population. Finally, the intestinal lymphatic system emerges as a critical conduit for disease propagation. Proteinuric kidney injury drives intestinal lymphangiogenesis, lymphatic dysfunction and compositional shifts in lymph (enriched in IsoLG–apoAI, inflammatory cells and cytokines), facilitating systemic dissemination of pathogenic mediators (167, 172). Therapeutic targeting of this axis encompasses three strategies: modulating VEGF-C/VEGFR3 signaling to suppress pathological lymphangiogenesis while preserving physiological drainage (173); deploying dicarbonyl scavengers (for example, 2-hydroxybenzylamine) to neutralize reactive lipid aldehydes such as IsoLG and block adduct formation (174); and engineering lymph-tropic nanoparticle or lipid formulations for site-specific anti-inflammatory delivery, thereby maximizing gut efficacy while minimizing systemic and renal off-target effects (175).

## 5 Conclusion and future perspectives

This review systematically elucidated the complex pathophysiological networks connecting IBD and renal disorders. We moved beyond viewing renal involvement as a mere extraintestinal manifestation, revealing that the kidney is a pivotal target organ in IBD within the framework of multiorgan network dysfunction. Injury mechanisms involve multilevel interplay, whereby dysregulation of the gut–kidney axis is manifested through biological crosstalk involving the microbiota, metabolites, and barrier function, and, as recent evidence highlights, through a distinct “gut–lymphatic–kidney” pathway. This newly delineated axis, fueled by kidney injury-induced intestinal lymphatic dysfunction and the systemic distribution of modified lipoproteins and inflammatory mediators via the lymph nodes, represents a novel paradigm for understanding systemic complications. This expanded understanding necessitates a shift from a single-organ focus toward an integrated multisystem perspective. We recommend increasing the prevention and monitoring of renal injury as a core component of standard IBD care and establishing a dynamic monitoring system based on risk stratification that integrates traditional markers with novel biomarkers and advanced imaging for the early detection of subclinical injury. Treatment decisions should comprehensively consider organ-specific and multisystem effects of medications, with particular attention paid to the coordinated management of shared complications.

Despite the progress reviewed in this review, significant gaps remain. First, the vast majority of mechanistic studies are derived from animal models; longitudinal human studies with serial renal biopsies, lymphatic imaging, and multi-omics profiling are urgently required to validate the pathways described. Second, biomarkers for early detection of subclinical renal involvement, particularly those reflecting lymphatic dysfunction or specific inflammatory axes, are lacking. Third, although microbiota-targeted therapies hold promise, their translation is hampered by the heterogeneity in donor selection, delivery methods, and safety concerns among immunocompromised populations. Fourth, the optimal frequency and modality of renal monitoring in patients with IBD remains poorly standardized, with no international consensus. Future research should focus on several frontiers to translate mechanistic insights into clinical breakthroughs. First, the causal roles of specific gut microbiota and metabolites within the gut–kidney axis and the identification of key biomarkers, potentially including lymphatic markers, for risk assessment and treatment prediction. Second, novel therapeutic strategies for gut–kidney comorbidity include precise microbial interventions, regulation of key inflammatory/fibrotic pathways (e.g., targeting IL-11 or GSDMD), investigation of multi-organ protective drugs (e.g., SGLT2 inhibitors), and pioneering lymphatic-targeted therapies (modulating lymphangiogenesis, scavenging dicarbonyls, and lymph-directed drug delivery). Finally, evidence-based multidisciplinary collaborative care models that integrate gastroenterology, nephrology, and nutrition should be established to develop individualized comorbidity management plans.

Overall, a deepening understanding of the IBD-renal disease association, now inclusive of the lymphatic vasculature as a key communication highway, is driving the transformation from symptomatic management to mechanism-targeted, multi-organ therapy. By integrating multiomics data, developing advanced disease models incorporating lymphatic function, and conducting targeted clinical trials, we can advance toward precision medicine for these comorbidities, ultimately improving long-term outcomes and quality of life. Advances in this field will benefit not only patients with IBD but may also provide insights into the systemic involvement of other chronic inflammatory diseases.
